# Biomimetic microenvironments for regenerative endodontics

**DOI:** 10.1186/s40824-016-0061-7

**Published:** 2016-06-02

**Authors:** Sagar N. Kaushik, Bogeun Kim, Alexander M. Cruz Walma, Sung Chul Choi, Hui Wu, Jeremy J. Mao, Ho-Wook Jun, Kyounga Cheon

**Affiliations:** Department of Biomedical Engineering, University of Alabama at Birmingham, Birmingham, USA; Department of Pediatric Dentistry, University of Alabama at Birmingham, SDB 311, 1720 2nd Ave South, Birmingham, AL 35294-0007 USA; Department of Pediatric Dentistry, Kyung Hee University, Seoul, South Korea; Center for Craniofacial Regeneration at Columbia University, New York City, NY USA

**Keywords:** Regenerative endodontics, Pulp-dentin tissue, Revascularization, Extracellular matrix, Biomimetic microenvironments, Tissue engineering

## Abstract

Regenerative endodontics has been proposed to replace damaged and underdeveloped tooth structures with normal pulp-dentin tissue by providing a natural extracellular matrix (ECM) mimicking environment; stem cells, signaling molecules, and scaffolds. In addition, clinical success of the regenerative endodontic treatments can be evidenced by absence of signs and symptoms; no bony pathology, a disinfected pulp, and the maturation of root dentin in length and thickness. In spite of the various approaches of regenerative endodontics, there are several major challenges that remain to be improved: a) the endodontic root canal is a strong harbor of the endodontic bacterial biofilm and the fundamental etiologic factors of recurrent endodontic diseases, (b) tooth discolorations are caused by antibiotics and filling materials, (c) cervical root fractures are caused by endodontic medicaments, (d) pulp tissue is not vascularized nor innervated, and (e) the dentin matrix is not developed with adequate root thickness and length. Generally, current clinical protocols and recent studies have shown a limited success of the pulp-dentin tissue regeneration. Throughout the various approaches, the construction of biomimetic microenvironments of pulp-dentin tissue is a key concept of the tissue engineering based regenerative endodontics. The biomimetic microenvironments are composed of a synthetic nano-scaled polymeric fiber structure that mimics native pulp ECM and functions as a scaffold of the pulp-dentin tissue complex. They will provide a framework of the pulp ECM, can deliver selective bioactive molecules, and may recruit pluripotent stem cells from the vicinity of the pulp apex. The polymeric nanofibers are produced by methods of self-assembly, electrospinning, and phase separation. In order to be applied to biomedical use, the polymeric nanofibers require biocompatibility, stability, and biodegradability. Therefore, this review focuses on the development and application of the biomimetic microenvironments of pulp-dentin tissue among the current regenerative endodontics.

## Background

Regenerative endodontics has been proposed to replace damaged and underdeveloped tooth structures with normal pulp-dentin tissue based on the concept of tissue engineering [1–3] by providing natural extracellular matrix (ECM) mimicking environment; stem cells, signaling molecules, and scaffolds [4–7]. Clinical success of the regenerative endodontics can be evidenced by absence of signs and symptoms; no bony pathology, a disinfected pulp, and the maturation of root dentin in length and thickness [6]. Current endodontic regeneration is often referred to as revascularization which disinfects the root canal using an antibiotic mixture and irritating the root apex tissue to form a blood clot inside the root canal to act as a natural scaffold and to support pulp-dentin stem cell proliferation and differentiation [6, 8, 9]. A blood clot can function as a scaffold for the ingrowth of new tissue since it consists of cross-linked fibrin [9, 10]. This is the pathway for migration of cells and helps with the growth and differentiation factors [5]. Biodegradable scaffolds have been developed to deliver dental mesenchymal stem cells [4, 11]. However, recent studies reported that the regenerated tissues from the revascularization are mainly dentin-like structure [12], cementum-like, and bone-like periodontal tissues [13–16]. Furthermore, the composition of the cells, signaling molecules, and scaffolds are not controllable to promote the pulp-dentin regeneration [6, 17]. Yet, there are still concerns about stem cell resources, required amount, transplantation, and immune responses [18]. Recently, the concept of cell homing has been developed by the recruitment of endogenous mesenchymal stem cells around pulp apex tissue [12, 19, 20]. There are still several macromolecules under investigation to recruit the endogenous pulp cells efficiently using chemo-attractants, ECM molecules [21], or platelet-rich plasma [22].

Despite the variety of approaches of regenerative endodontics, there are several major challenges that remain: (a) The endodontic root canal is a strong harbor of the endodontic bacterial biofilm and the fundamental etiologic factors of recurrent endodontic diseases; therefore, effective disinfection is critical for the success of pulp-dentin regeneration [6]; (b) Tooth discolorations were caused by minocycline (MC) [23] from the triple antibiotic mixture [8, 24] or mineral trioxide aggregates (MTA) [25]; (c) Cervical root fractures were reported due to the calcium hydroxide (Ca(OH)_**2**_) [26–28]; (d) The scaffold should be biocompatible and biodegradable [29, 30]; And (e) the pulp-dentin complex should be highly vascularized with innervated pulp as well as a dentin matrix with adequate root thickness and length [4, 6]. Overall, the current clinical protocols have limited success in the regeneration of the pulp-dentin tissue.

Tissue engineering is the application of life sciences and biomaterials engineering for the development and advancement of tissue mimicking structures and the function of their natural counterparts [1, 31]. Existing cells, biomaterials, and the oral cavity’s natural chemistry will be utilized to synthesize a natural-like microenvironment. Therefore, this review focuses on the development and application of the biomimetic microenvironments of pulp-dentin tissue among the current regenerative endodontics.

## Review

### Anatomy of pulp-dentin complex

**Fig. 1:**
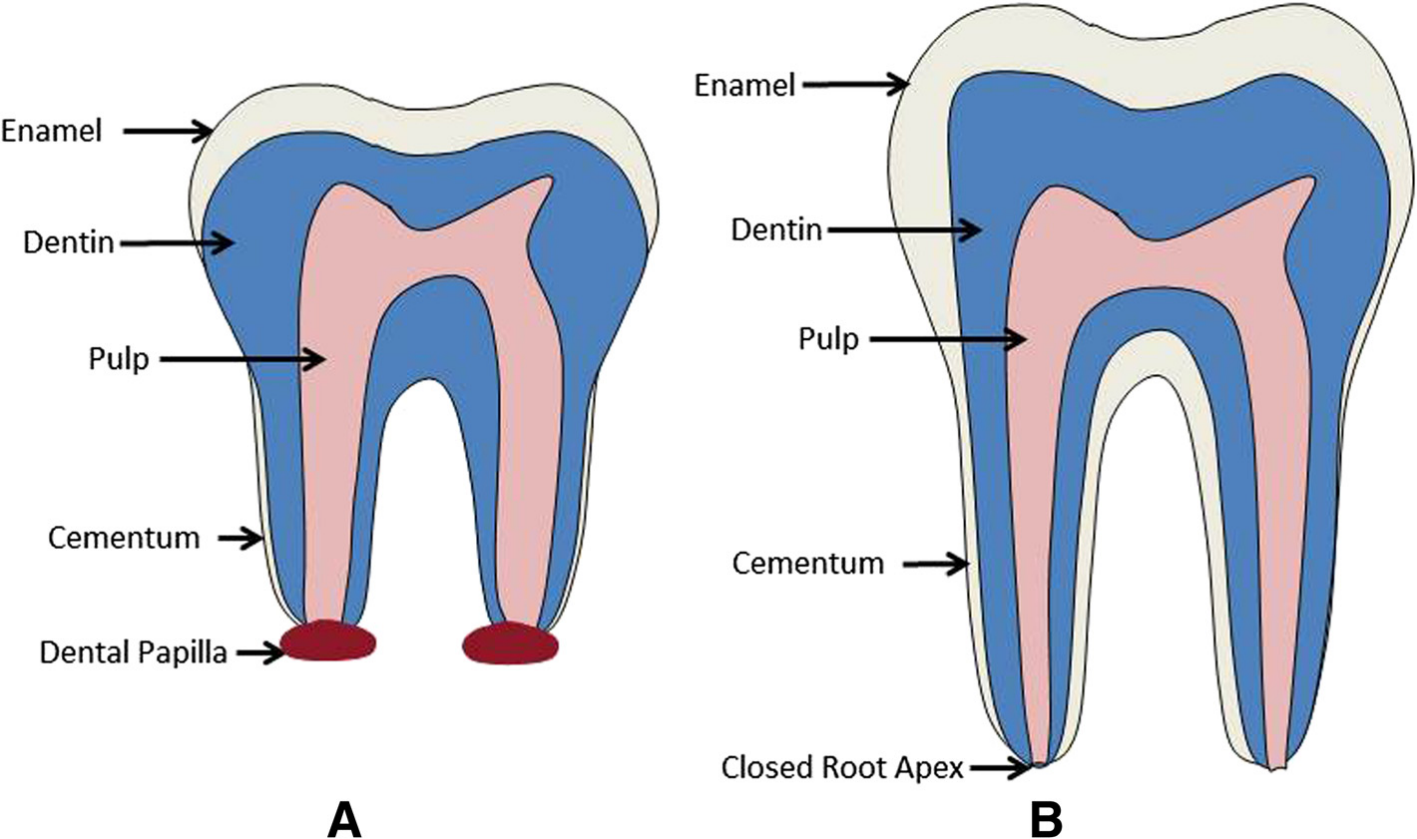
Anatomy of tooth; (**a**) a healthy immature tooth with the distinct open root apex surrounded by dental papilla. **b** a healthy mature tooth with a closed root apex

The dental pulp is comprised of loose connective tissues originated from the dental papilla of the tooth germ and their close proximity and interdependence cause the formation of the pulp-dentin complex separated by the outer layer of the dental papilla (odontoblast layer) [32]. Dentin and pulp tissue are confined with enamel tissue, which is not exposed on oral cavity; thus the proper understanding of the pulp-dentin complex is crucial for the development and progression of microenvironment-based regeneration. Mature dentin is a mineralized form of the collagen-based predentin matrix and its crystalline structure primarily consists of hydroxyapatite and water that surrounds the dental pulp [33]. The pulp consists of pulp cells, odontoblasts, endothelial cells, neurons, immune system cells, and the ECM, which is crucial in maintaining the function of healthy teeth [34, 35]. The apical foramen of the tooth allows for nutrients to be supplied and waste to be excreted through blood vessels [36]. Figure 1 demonstrates the characteristics of an immature tooth having an open apex, large canal, and a short root, which make the new tissue easily develop into the root canal space. Further the new tissue ultimately is regenerated into the coronal pulp chamber [37], which will promote revascularization and reinnervation [11]. On the other hand, a permanent tooth with a mature apex with a small canal may only have a limited amount of blood supply to allow ingrowth of tissue into the root canal space [38].

### Root end closure (Apexification)

Steps for current revascularization for necrotic immature teeth involve opening the root canal and disinfecting with sodium hypochlorite (NaOCl). Lower concentrations (1.5 %) of NaOCl and saline are used with an irrigating needle positioned about 1 mm from root end, to minimize cytotoxicity to stem cells in the apical tissues [39, 40]. Then, the area of the root canal is filled with a triple antibiotic paste, consisting of ciprofloxacin, metronidazole, and minocycline with inactive carriers (Macrogol ointment and Propylene glycol) [24, 37], for one to four weeks and sealed with temporary restorative material such as Cavit™, IRM™, glass-ionomer or another temporary material [39]. At the consequent follow up, the treated root canal is accessed to remove the antibiotic paste upon the re-evaluation of the signs and symptoms, irrigated with 17 % Ethylenediaminetetraacetic acid (EDTA) to release growth factors from the dentin [41], and the root apex is stimulated to form a blood clot into canal by over-instrumenting endodontic files and bleeding is confined at the level of cemento-enamel junction avoiding tooth discoloration. A resorbable matrix (CollaPlug™, Collacote™, or CollaTape™) is placed on top of the blood clot, then sealed with a MTA with/without Ca(OH)_2_ as a capping material [39, 42–45]. Several challenges were reported from the apexification procedure of immature necrotic teeth [42].

### Challenges for apexification procedures

#### Discoloration of tooth

One of the main problems from the use of the current protocol is the discoloration of the tooth crown due to the use of tetracycline (e.g. minocycline) in the triple antibiotic mixture [24, 46, 47]. Therefore, minocycline was replaced with other equivalent antibiotics in recent studies (e.g. cephalosporin, amoxicillin etc.,) resulting in no further discoloration [42, 48, 49]. Other studies showed that the combination of metronidazole and ciprofloxacin with any of these antibiotics was just as effective in sterilizing carious and endodontic lesions [49]. Another solution to avoid the tooth discoloration can be observed in a modified protocol by sealing the dentinal tubules using MTA below the gingival margin [23, 47]. Along with the sealing dentinal tubules, intra-coronal bleaching with sodium perborate using white MTA instead of grey MTA is also suggested [23].

#### Cervical root fracture

Traditionally, Ca(OH)_2_ was used for the apexification procedure of the immature root with pulpal necrosis as the intra-canal medicament [50]. In spite of the reported clinical success, there are potential complications for the traditional protocol [27, 51]. Due to its high pH, calcium hydroxide can cause necrosis of tissues that could potentially differentiate into new pulp. Apexification procedures can leave the immature tooth fragile because the root remains short with thin, radicular walls, making the tooth more susceptible to fracture [51]. In cases disinfected by calcium hydroxide, root canal calcification/obliteration was observed [52–54]. Studies conducted by Andreasen and other researchers have demonstrated that the traditional use of long-term application of Ca(OH)_2_ can lead to a weaker tooth more susceptible to fracture [27, 55, 56]. In addition, Ca(OH)_2_ procedure requires a long treatment period for the formation of the calcified barrier from 3 to 24 months with multiple applications [50, 57].

#### Creation of blood clot

In the current protocol, blood clot is created by over-instrumenting beyond the root apex to provide scaffold inducing source of growth factors and repairing pulp tissue [9, 42, 58, 59]. The induced blood clot may serve as a natural scaffold to allow the migration of stem cells along the canal [8, 38]. However, the inability to consistently produce an ideal blood clot was also observed [42] and limited tissue regeneration was observed. Absence of a blood clot would hinder such a migration, which may be caused by the vasoconstrictor epinephrine in the local anesthetic solution [38, 42]. To resolve the issues, local anesthetic without a vasoconstrictor can be chosen [42]. Meanwhile, there are concerns for the stimulated pulp bleeding which may not be the ideal procedure or function as a scaffold to induce the pluripotent stem cells resulting uncertain pulp-dentin tissue regeneration [4, 20].

#### Poor root development

Ideal root development pattern in immature teeth would include an increase in root length and wall thickness with formation of the root apex [15]. However, tooth necrosis followed by regenerative endodontic treatments has been reported to have an absence of increase in root length and root wall thickness, or a lack of tooth apex formation [54, 60, 61].

A retrospective evaluation of radiographic outcomes discovered that regenerative endodontic treatment with triple antibiotic dressing increased root length more than MTA apexification and root wall thickness significantly more than either Ca(OH)_2_ or formocresol [62]. Yet, the replaced structures were found to be periodontal tooth structures such as cementum-like, bone-like, or fibrous connective tissue structures by histologic sections [13–15, 63]. Yamauchi et al. attempted to improve dentin formation through the use of a cross-linked collagen scaffold in the canal spaces of dogs with apical periodontitis. The results showed the formation of distinct mineralized tissues, dentin-associated mineralized tissue (DAMT) and bony islands (BIs) [64]. Through immuno-histochemical analysis, it was determined that DAMT resembled cementum without any vasculature. The BIs were found to resemble bone because it was vascularized with lacunae and “bone marrow-like” structures [65]. However, there was no evidence of pulp-like tissue or dentin-like structures in any of specimens, which are the key components in endodontic tissue regeneration. Yamauchi et al. recommend the incorporation of “some factors into the scaffold that facilitate the differentiation of stem cells to odontoblasts” in order to create the pulp-dentin complex [64].

#### Cytotoxicity

Intracanal medicament, antibiotics can induce cytotoxic effect on dental pulp stem cells [49]; it can be due to the lowered pH from the antibiotics, minocycline hydrochloride and ciprofloxacin hydrochloride (HCl), which are used in the triple antibiotic mixture. The release of hydrogen ions from HCl groups resulted in an acidic condition, which can be an unfavorable condition for culturing cells [66]. Conversely, recent in vitro cytotoxicity studies demonstrated that metronidazole did not inversely affect human dental pulp cells (DPCs) and apical papilla cells (APCs) even at the 25.00 mg/mL concentration. Metronidazole solution may have a neutral pH, which can explain why cytotoxicity did not occur [67]. On the other hand, the triple antibiotic at 0.39 mg/mL had a less cytotoxic effect on DPCs and APCs viability [67]. The single antibiotic with the concentrations of 0.024 μg/mL maintained dental pulp cell viability for 7 days [67]. Also, lower than 2.5 mg/mL of the triple antibiotic and Ca(OH)_2_ demonstrated no cytotoxicity on the DPCs using lactate dehydrogenase activity assay [68]. Therefore, the concentration of triple antibiotic in clinical usage suggested to be adjusted not to cause cytotoxicity on the remaining vital tissues.

### Development of regenerative endodontic procedures (REP)

Tooth development is the multistage process between oral epithelium and mesenchymal origin, resulting in the formation of the dentin matrix and pulp-dentin complex. Ectomesenchymal stem cells from dental papilla differentiate into dentin-forming odontoblasts [69, 70]. Hertwig’s epithelial root sheath (HERS) from the inner and outer enamel epithelium are critical components in the process since they guide the underlying mesenchymal cells from the dental papilla and follicle to differentiate into odontoblast, pulp fibroblast, and cementoblast of the root [14]. Through this development, root dentin would increase in length and thickness.

In a study conducted by Murray et al., the researchers used the term “regenerative endodontic procedures” (REPs), which is a ‘biologically based procedure designed to replace damaged structures’ such as root dentin along with cells of the pulp-dentin complex [2]. The goal in REP is to provide a suitable environment in the root canal that will promote repopulation of the osteo/odonto progenitor stem cells, regeneration of pulp tissue, and continued root development [36]. Endodontic treatment utilizing osteo/odonto progenitor stem cells in the apical papilla is resistant to the infection and necrosis caused by proximity to periodontal blood supply [38].

REP has been shown to have distinct differentiation potential using mesenchymal stem cells markers [39, 71]. In a study conducted by Hristov et al., blood vessels were identified through the use of double-immunostaining for CD31/collagen-IV and Vascular endothelial growth factor (VEGF)R2/Collagen-IV; the process of revascularization was occurring in the endothelial progenitor cells during their differentiation [72]. Despite the lack of REP-associated clinical trials, clinicians continue to use this method for treatment. The American Association of Endodontics (AAE) commented on this controversy and said that regenerative endodontics is ‘one of the most exciting new developments in dentistry today.’ After this, the AAE developed treatment considerations and asked practitioners to use this approach while keeping the new research findings in mind [39, 73]. Therefore, the REP may provide a sufficient disinfection and influence cell survival, migration, angiogenesis, proliferation, and differentiation [62].

#### Revascularization or Revitalization

The formation of blood vessels around the teeth that provide blood supply in teeth is known as vascularization, which is important in tooth development and function [8]. Therefore, the term “revascularization” was coined from a case report describing the re-establishment of blood supply in teeth with incomplete root formation after an auto-transplantation or replantation. In a study conducted by Iwaya et al., revascularization was suggested to treat an immature permanent tooth with ‘apical periodontics and sinus tract,’ as an alternative procedure to apexification [26, 74]. As demonstrated by Kling et al., successful regeneration is dependent on the rates of formation of new tissues versus the bacterial growth. If the radiographic opening is more than 1.1 mm, the incidence of revascularization is enhanced. As a part of the revascularization treatment, a blood clot is created after the canal is disinfected to act as a matrix for the growth of new tissue in the space [75]. Banchs and Trope used a double seal with MTA and bonded resin to prevent any bacteria from invading the pulp space before the revascularization could occur [8]. Along the same lines, “revitalization” is a term that describes an endodontic procedure used to rejuvenate tooth vitality in the case of necrotic stages; “regeneration” in endodontics has been defined as procedures of replacing lost or damaged pulp-dentin tissues complex [2]. However, histological studies show that the tissue found in root canals may not be through the exact regeneration process, but instead through healing process which is known as “repair”. The repair of the tissue has been used when the healed tissue inside the root canal recovers the similar form and elements of pulp tissue [76].

### Bioengineering approaches for REP

#### Dental stem cells

The fields of stem-cell based pulp-dentin regeneration along with cell-free approaches have been developed. Recently, a new population of mesenchymal stem cells (MSCs) has been discovered stem cells from the apical papilla (SCAP) of immature teeth and stem cells from human exfoliated deciduous teeth (SHED) derived from pulp tissue or the precursor of pulp [77–79]. They have been shown to be distinct from dental pulp stem cells (DPSCs) through histologic, immunohistochemical, cellular, and molecular analyses [80], and seem to be responsible for dentin formation in the root [38]. Autologous DPSCs with growth factor, bone morphogenetic proteins (BMP) 2 has successfully shown partial pulp regeneration in a dog model [81]. Furthermore, DPSCs was shown to produce neurotrophic factors to induce neural tissue development [77, 82]. Besides SCAP, which has shown promising pulp regeneration capability, subpopulations of pulp stem cells, bone marrow MSCs (BMMSCs) and adipose tissue-derived MSCs (ADMSCs) also can regenerate pulp tissue [83]. A growing amount of evidence is demonstrating that SCAP is the source of the primary odontoblasts for the formation of the root dentin, whereas DPSCs are the source of replacement odontoblasts. Critical roles of the SCAP for the continued root formation are highlighted [38] and the SCAP and other type of stem cells (e.g. periodontal ligament stem cells) can be combined for the root regeneration [78]. In order to evaluate the regenerative potential, DPSCs and SCAP were encapsulated into a scaffold and inserted into section of human tooth root canal and transplanted into severe combined immunodeficiency mice subcutaneously for three to four months; as a result, pulp space was filled with vascularized pulp-like tissue and uniform dentin-like layer at dentin wall and MTA cement [11]. Therefore, a stem cell based engineering approach can provide realistic pulp-dentin regeneration. In addition, vascularization is a critical component of pulp-dentin regeneration, which can be accelerated with several angiogenic factors; VEGF and platelet-derived growth factor [84–86].

#### Nitric oxide

Angiogenesis is an important process that is required for many pathological and wound healing processes. VEGF is an inducer of angiogenesis that promotes the vessel formation. Nitric oxide (NO) is a lipophilic molecule that can easily permeate biological membrane barriers and has been found to be a potent vasodilator [87] and the amount of NO can also regulate VEGF [88]. In addition, NO releasing dendrimers are reported as effective antibacterial agents [89, 90]. They tested a series of NO-releasing poly (propylene imine) (PPI) dendrimers and control PPI dendrimers (non-NO-releasing) against Gram-positive and Gram-negative pathogenic bacteria. It was found that the NO-releasing PPI dendrimers killed > 99.99 % of all bacterial strain tested with a minimal toxicity to mammalian fibroblasts [89]. Through this dual function of NO, NO releasing scaffolds can be utilized in REP and other tissue engineering fields.

#### Bone morphogenetic proteins

Bone morphogenetic proteins (BMPs) have been implicated in tooth development, and the expression of BMP2 is increased during the terminal differentiation of odontoblasts [91, 92]. Beads soaked in human recombinant BMP2 induce the mRNA expression of dentin sialophosphoprotein (DSPP), the differentiation marker of odontoblasts and indication of producing of dentin matrix proteins after implantation onto dental papilla in organ culture. BMP2 also induces a large amount of reparative dentin on the amputated pulp in vivo [93]. BMP2 may play a role in regulating the differentiation of pulp cells into odontoblastic lineage and also stimulate reparative dentin formation [92].

#### Enamel-like fluorapatite surfaces

Previous studies have also demonstrated good biocompatibility of both the ordered (OR) and disordered (DS) Fluorapatite (FA) crystal surfaces in providing a favorable environment for functional cell-matrix interactions of human DPSCs [94, 95]. In addition, studies have shown long-term growth of human DPSCs. Specifically, enhanced cellular response of DPSCs to the OR FA crystal surface has been observed [95, 96]. This can be further manipulated by treating with dentin-inducing-supplement to produce a dentin/enamel superstructure [94, 95]. Studies have shown that FA crystal surfaces, especially the OR FA surface, indeed can and did mimic the physical structure of enamel and also provided a favorable extracellular microenvironment for the cells [95, 96]. Furthermore, FA crystal surfaces induced and stimulated differentiation of human DPSCs and mineralization of tissue formation without a mineralization supplement. Such findings display the promising benefits of utilizing FA crystal surfaces as a simple biomimetic model for dentin regeneration, enamel/dentin/pulp complex creation, and also as a scaffold for hard tissue engineering [96].

#### Platelet-rich plasma

Platelet-rich plasma (PRP) contains multiple growth factors, which include platelet-derived growth factor, transforming growth factor b, and insulin-like growth factor [97]. Thus, PRP may be a good supplement for cell-based pulp/dentin regeneration. PRP, which can be derived from a patient’s own blood, is easy to prepare and can also form a three-dimensional fibrin matrix that can act as a scaffold [36, 98, 99]. An in vitro study showed that PRP can enhance the proliferation and differentiation of human DPSCs [100]. In the present study, only PRP or the combination of PRP and DPSCs did not enhance the true regeneration of necrotic tissue rather stimulate tissue repair with newly formed cementum like, bone like, and connective tissues [101]. Another collagen scaffold used by Iohara et al. to carry DPSCs into the canals may provide a better condition for pulp regeneration compared [102]. The in vitro study showed that, although PRP can enhance mineralization differentiation of DPSCs, it is not clear whether PRP enhances dentinogenesis (i.e., PRP may not promote pulp-dentin regeneration) [100].

#### Cell homing

Some researchers have also seen positive results of the regeneration of pulp-like tissue through chemotaxis induced cell homing [12, 19, 20]. The cell homing is a process, migration of mobilized hematopoietic stem cells via vascular structure toward certain tissues (e.g. any organs, injured tissues) using active navigation [103–105]. This concept leaves potential pulp-dentin re-cellularization and revascularization with or without active apical papilla tissue. A variation of pulp-dentin regeneration can be resulted from the combination of cell homing with cell transplantation and a variety of the growth factors [76]. Therefore, the migrated SCAP in periapical tissues is reported to have a positive role to be differentiated into pulp-dentin forming cells [38, 78]. However, the migrated MSCs in periapical tissues may form ectopic periodontal tissue in the pulp space [13, 14]. Besides, BMSCs are also considered for migrated source of forming pulp tissue [106]. During the homing process, various growth factors play a critical role to assist stem cells; for example, BMP7 was delivered to promote the regeneration of dentin-like tissue and create an ideal microenvironment [12]. Stem/progenitor cell-based approaches are also being studied by researchers. Stem/progenitor cells from apical papilla and DPSCs were isolated and seeded onto a synthetic porous scaffolds consisting of poly-D, L-lactide and glycolide [107]. Subsequently, dentin-like tissue was observed expressing by dentin sialophosphoprotein, bone sialoprotein, alkaline phosphatase, and CD105 as would their natural counterparts [107].

#### Biomimetic microenvironments

To regenerate the function and form of the pulp-dentin complex, the construction of the biomimetic microenvironment is a key factor. Cells respond differently to physicochemical and mechanical properties of the microenvironment. The interactions between cells and the ECM control differentiation, migration, and proliferation, as well as tissue remodeling. For this reason, an ECM mimicking microenvironment has been designed by incorporating various moieties and features derived from the ECM. Biomimetic environments, such as ECM microenvironments through peptide amphiphiles (PA), cell homing, stems cells and through growth factors, have been developed [1, 8, 26, 107].

**Fig. 2:**
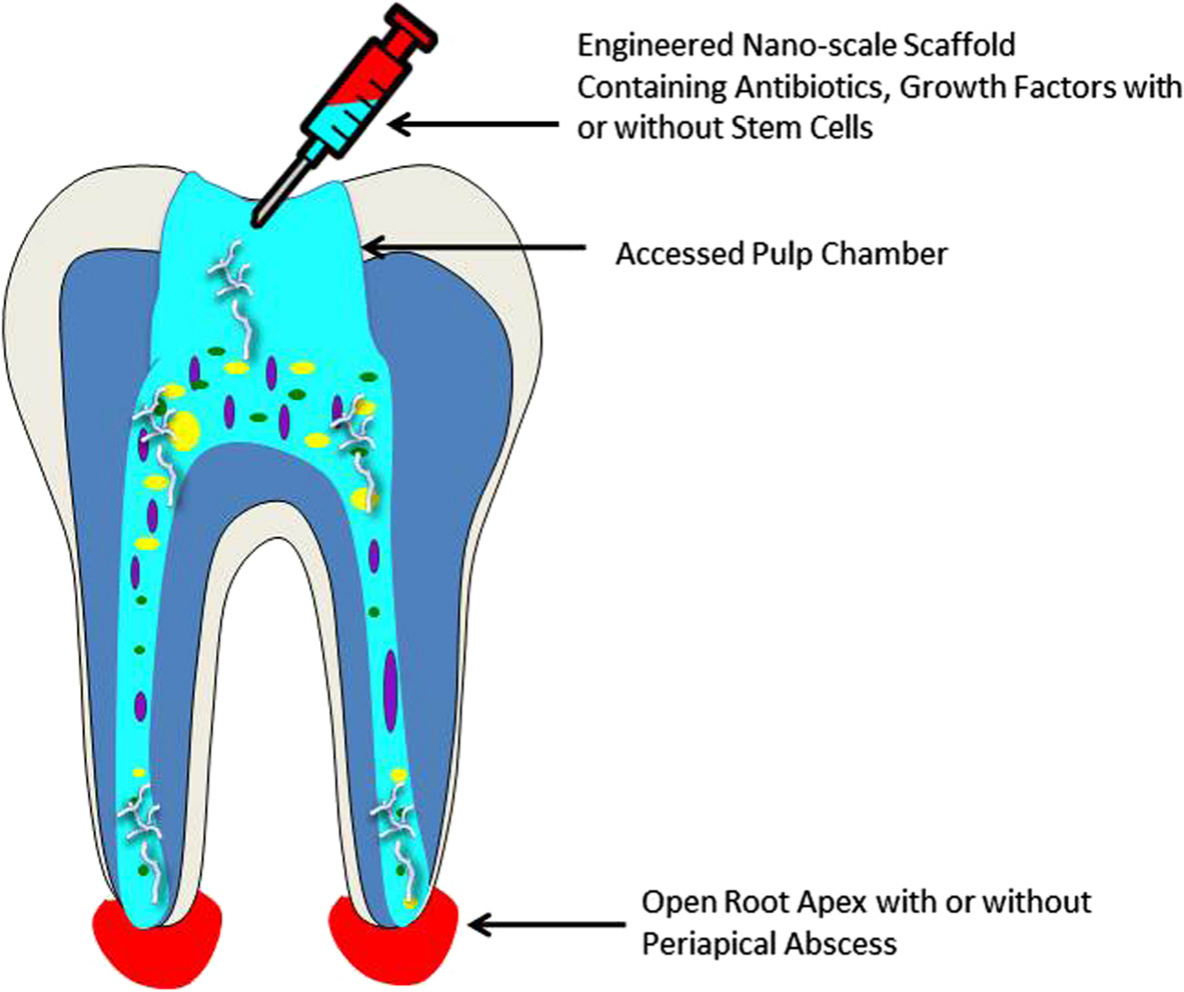
Engineered nano-scale scaffold for the regenerative endodontics treatment for an infected tooth; after removal of infected pulp-dentin tissue; the root canal is irrigated with NaOCl and EDTA. Engineered nano-scale scaffold containing a mixture of antibiotics, growth factors, and/or stem cells is applied to the root canal

**Fig. 3:**
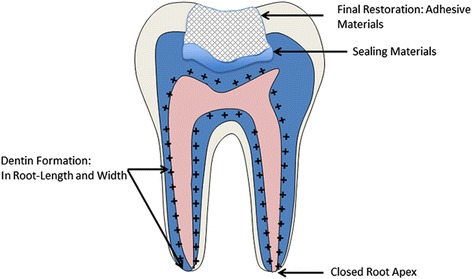
Regenerated pulp-dentin tissue with closed root apex; regenerated pulp-dentin tissue with closed root apex is observed after the regenerative endodontic treatment using an engineered nano-scale scaffold. Removed coronal structure is restored with adhesive materials with base sealing materials. Plus (+) signs indicate the area of dentin formation

ECM proteins potentially carry problems for clinical applications including undesirable immune responses, higher risks for infection, variety in biological sources, and increased clinical costs [108]. To overcome such limitations, small peptide sequences derived from ECM proteins have been utilized such as Gly-Arg-Gly-Asp-Ser (GRGS) [109, 110]. However, these isolated ECM peptides still possess some limitations of encapsulating biomaterials. For example, after implantation, entrapping cells in photo-polymerized biomaterials can potentially have many problems, such as the formation of fibrotic processes, poor degradation of the scaffold, and local and/or systemic toxicity [111]. Studies have also shown that different compositions and concentrations of alginate can affect the cellular overgrowth of implanted capsules. This can be due to the formation of the metabolic barriers to nutrient diffusion around the implant if inadequate levels of the material are used [112]. To overcome such limitations, nano-scale PA nanomatrix gels [113] have been proposed as a promising solution by synthetically recapitulating the ECM structure as shown in Figs. 2 and 3. PA nanomatrix gel possesses such qualities: rapid gel-like 3D network formation by self-assembly, versatility to incorporate various cell adhesive moieties, and cell-mediated degradable sites (matrix metalloproteinase-2) for progressive scaffold degradation and eventual replacement by host-ECM [114].

**Fig. 4:**
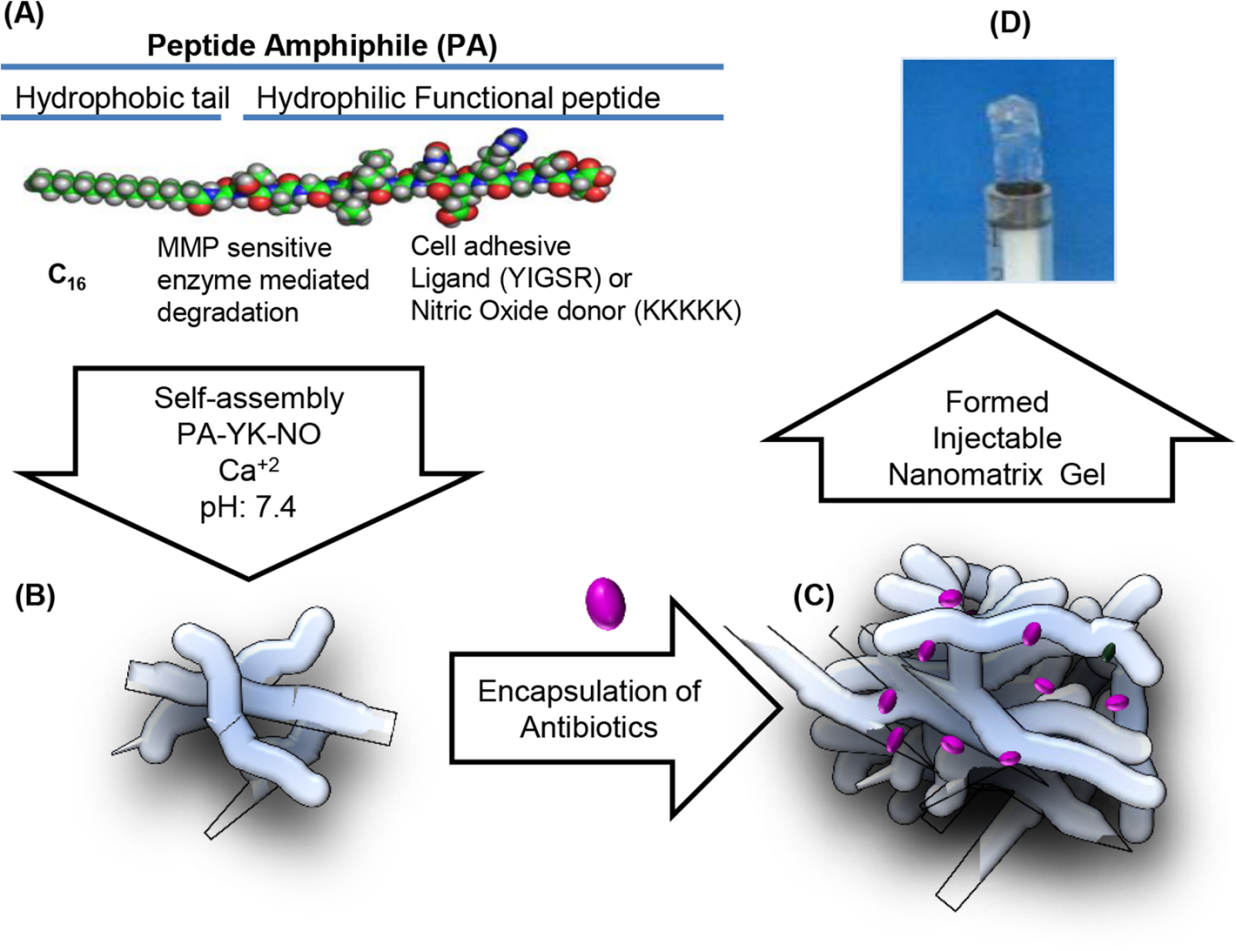
General scheme of the design for the biomimetic approach; (**a**). Synthesis of peptide amphiphiles (PAs), (**b**). Self-assembly of PAs, (**c**). Encapsulation of antibiotics, (**d**). Formation of the nanomatrix gel, Modified with permission from Kaushik et al. [119]

The PA is a hydrophilic head, consisting of a functional peptide sequence, attached to a hydrophobic alkyl tail. The internal peptide structure can be modified to mimic the characteristic properties of the natural ECM [115–118]. Furthermore, PA self-assembles into long cylindrical structures which are 8–10 nm in diameter and up to several microns in length. As seen in Fig. 4, Kaushik et al. have developed a biomimetic antibiotic releasing nanomatrix gel that demonstrates synergistic antibacterial effects, which may be effective for root canal disinfections and eliminates the use of minocycline, which is used in the traditional protocol [119]. The development of the gel, which uses PA for the encapsulation of the antibiotics to create a sustained local release drug delivery system, is still in preliminary stages but shows very promising results in early studies. The developed gel, which contains ciprofloxacin and metronidazole, was tested against two prominent bacterial strains in endodontic infections*, E. faecalis* and *T. denticola*. Their results portrayed that the developed gel had a greater synergistic antibacterial effect than the antibiotics alone [119].

### Animal models for microenvironment viability

Recently, there are DPSCs that have been used in both small and large animals, which demonstrate that pulp or dentin like tissues are able to regenerate either partially or completely for the root canal space [84]. An experimental animal model is required with comparable “anatomical, physiologic, histologic, and pathologic characteristics to the ultimate treatment cohort [120].” This means that the animal model should have relatively large teeth that are easily accessible and able to be radiographed. It is also preferred that the model be inexpensive and readily available. The advantages and disadvantages of various animal models, including rats, cats, ferrets, dogs, and primates are discussed.

#### Rats

Rats and mice are the preferred animal model in many fields of research because they are inexpensive, convenient, and well understood. They are convenient because they are small and easily maintained, and can be bought in relatively large quantities for low prices. Unfortunately, rodents’ teeth are too small for experiments in regenerative endodontics, though they have been used successfully in studies regarding pulp and periapical tissue reactions [121]. Similarly, guinea pigs and rabbits have teeth that are simply too small for endodontic regenerative studies. A study conducted by Zhao et al. used transplanted rat teeth, and demonstrated that in some cases there was revascularization of the pulp, and dentin-like structures were able to form on the root wall [122]. This auto-transplantation study may provide insight into the biological process of the regeneration of the pulp-dentin complex.

#### Ferrets

Numerous areas of research have utilized the ferret including neuroscience, pathogenesis, endocrinology, and the study of numerous diseases. However, the ferret has not been used extensively in the field of endodontics. Due to the accessibility and larger size of the ferret’s single-rooted cuspid, the ferret is more suitable for endodontic regenerative studies than rodents and rabbits. Additionally, the ferret is subject to less ethical objections than dogs, cats, and primates as well as being more readily available and less expensive [123]. A study in 2011, conducted by Torabinejad et al., investigated into the use of ferret cuspid as a model for regenerative endodontics using radiography [120]. It was determined that a ferret’s cuspid teeth erupted around 50 days after birth with open apices. At 52 days, the HERS “was extending to form the root, with very thin walls, a wide canal space, and an open apex” [120]. Apical closure began at approximately 90 days, continuing until complete closure observed at 133 days. The study concluded the most appropriate time to conduct studies on ferret teeth is during the 50–90 days when the open apex allows communication between the root canal system and the periapical tissue. Torabinejad et al. stress that more research into the ferret model is required, with the need for the development of a stem cell population in the ferret pulp and periapical tissues, in addition to the development of specific antibodies that can decisively identify relevant dental tissues [120].

#### Cats

Cats can provide four large single-rooted cuspids that are similar in craniofacial characteristics to humans. Wilson found that all permanent teeth before the age of six months are erupted and have open apices, with closure of cuspids occurring approximately at nine months, and complete closure at eleven months [124]. Cats are relatively expensive to purchase and maintain. Additionally, there has been an increase in public objection to the use of cats in research because they are common domesticated pets in numerous cultures.

#### Dogs

Dogs have been used in various endodontic researches, including regenerative studies [13, 64, 125, 126]. Apices of permanent teeth remain open until 6 months of age and will be closed at 10 months old [127]. Khademi et al. used single-rooted premolars and maxillary incisors from 3 immature mongrel dog’s to induce periapical lesions for the evaluation of the success rate of a revascularization treatment protocol [125]. Mandibular incisors were deemed unsuitable due to their susceptibility to fracture under large masticatory forces, and the apex closes before sufficient dentinal wall can develop. In addition, the proximity of mandibular roots makes it difficult to take clear radiographic images. Induced necrotic-infected teeth can develop periapical lesions after about 28 days. In the dog model, the “dental pulp tissue possesses a capacity for spontaneous repair by the formation of reparative dentin, but only up to a defect size of 2 mm in diameter and 1 mm in depth [126].”

#### Primates

Primates, being the closest ancestor to humans, are the ideal animal models for a lot of medical and dental research [121]. Although longitudinal studies on the age of eruption and root end closure in different species of primates are unavailable, Anemone et al. studied apical closure radiographically in chimpanzees. Although primates are the most similar to humans, they are not used extensively due to their high cost to purchase and maintain in addition to the difficulty of handling. There are also the ethical problems that come with the fact that primates are so similar to humans.

## Conclusions

This review article is focused on the current prospects on biomimetic microenvironments as a scaffold of pulp-dentin complex regeneration via current tissue engineering concepts. The proper biomimetic microenvironments can be constructed upon the synthetic nano-scaled peptide amphiphiles through bioengineered regeneration process in combination with various bioactive molecules, growth factors, and stem cells to mimic native pulp ECM. From the animal models, currently the dog model is favorable to perform regenerative endodontic studies due to its availability and similarity of the size and number of teeth for the creation of a biomimetic microenvironment. In spite of the promising data from in vitro and some animal experiments, the future advances in pulp-dentin tissue regeneration are required to show the functional tissue regeneration in addition to the favorable clinical outcomes.

## Abbreviations

AAE, American Association of Endodontists; ADMSCs, adipose tissue-derived MSCs; APCs, apical papilla cells; BIs, bony islands; BMMSCs, bone marrow MSCs; BMPs, bone morphogenetic proteins; Ca(OH)_2_, calcium hydroxide; DAMT, dentin-associated mineralized tissue; DPCs, dental pulp cells; DPSCs, dental pulp stem cells; DSPP, dentin sialophosphoprotein; ECM, extracellular matrix; EDTA, ethylenediaminetetraacetic acid; FA, fluorapatite; HCl, hydrochloride; HERS, Hertwig’s epithelial root sheath; MC, minocycline; MSCs, mesenchymal stem cells; MTA, mineral trioxide aggregates; NaOCl, sodium hypochlorite; NO, nitric oxide; PAs, peptide amphiphiles; PPI, propylene imine; PRP, platelet-rich plasma; REP, regenerative endodontic procedures; SCAP, stem cells from the apical papilla; SHED, stem cells from human exfoliated deciduous teeth; VEGF, vascular endothelial growth factor.
